# Disease-Associated Circular RNAs: From Biology to Computational Identification

**DOI:** 10.1155/2020/6798590

**Published:** 2020-08-17

**Authors:** Min Tang, Ling Kui, Guanyi Lu, Wenqiang Chen

**Affiliations:** ^1^School of Life Sciences, Jiangsu University, 301 Xuefu Road, Zhenjiang, Jiangsu 212013, China; ^2^Dana-Farber Cancer Institute, Harvard Medical School, Boston, MA 02215, USA; ^3^Beijing Institute of Pharmacology and Toxicology, 27 Taiping Road, Beijing 100850, China; ^4^Section on Integrative Physiology and Metabolism, Joslin Diabetes Center, Harvard Medical School, 1 Joslin Place, Boston, MA 02115, USA

## Abstract

Circular RNAs (circRNAs) are endogenous RNAs with a covalently closed continuous loop, generated through various backsplicing events of pre-mRNA. An accumulating number of studies have shown that circRNAs are potential biomarkers for major human diseases such as cancer and Alzheimer's disease. Thus, identification and prediction of human disease-associated circRNAs are of significant importance. To this end, a computational analysis-assisted strategy is indispensable to detect, verify, and quantify circRNAs for downstream applications. In this review, we briefly introduce the biology of circRNAs, including the biogenesis, characteristics, and biological functions. In addition, we outline about 30 recent bioinformatic analysis tools that are publicly available for circRNA study. Principles for applying these computational strategies and considerations will be briefly discussed. Lastly, we give a complete survey on more than 20 key computational databases that are frequently used. To our knowledge, this is the most complete and updated summary on publicly available circRNA resources. In conclusion, this review summarizes key aspects of circRNA biology and outlines key computational strategies that will facilitate the genome-wide identification and prediction of circRNAs.

## 1. Introduction

Circular RNAs (circRNAs) are traditionally viewed as noncoding RNAs that form a covalently closed continuous loop and thought to be generated from imperfect splicing. However, emerging evidence has shown a complexity of circRNAs in gene expression regulation, and thus the notion that circRNAs are of low abundance has been gradually challenged. Thus, the generation of circRNAs from such noncanonical RNA splicing appears to be a feature of human gene expression [1].

**Figure 1:**
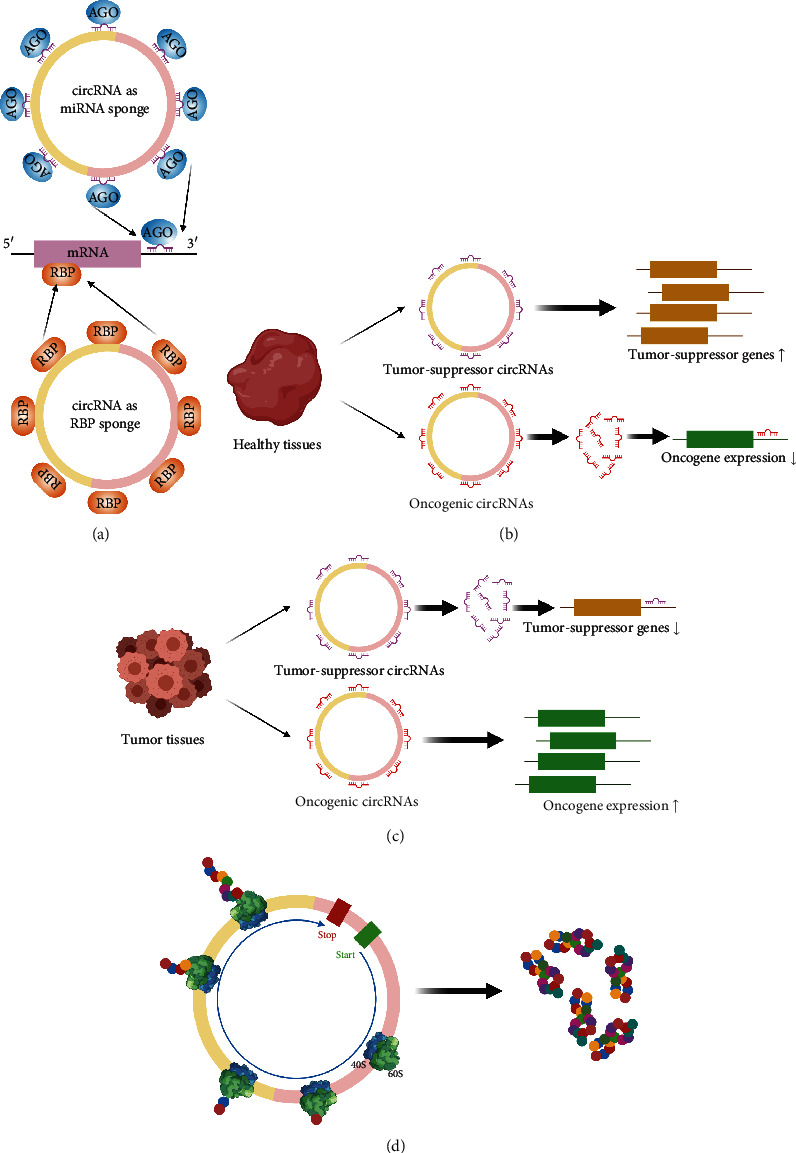
Translation of circRNAs. (a) circRNAs can serve as a miRNA sponge, containing multiple binding sites for miRNAs (blue) or RBPs (in red), thus affecting gene regulation. (b and c) Illustrations show the role of circRNAs as miRNAs in healthy and tumor tissues. Tumor-suppressor circRNA sponges contain binding sites for tumor-suppressor miRNAs (light purple), while oncogenic circRNA sponges contain binding sites for oncogenic miRNAs (red). Tumor-suppressor circRNAs upregulate tumor-suppressor genes (yellow) in healthy tissues but downregulate these genes in tumor tissues, whereas oncogenic circRNAs suppress oncogene (green) expression in healthy tissues but upregulate these genes in tumor tissues. AGO: Argonaute; RBP: RNA-binding protein. Illustration is inspired by and modified from [164]. (d) New studies suggest that circRNAs generated by backsplicing are able to be translated into proteins. Illustration is modified from [38, 39]. Illustrations were generated using BioRender.

Recent evidence has shown that circRNAs can act as microRNAs (miRNAs) and protein sponges as well as regulators for translation and posttranslation (Figure 1) [2–5], although other functions are also reported [6, 7]. Contemporaneous studies have revealed that dysfunction of circRNAs is closely linked to a broad range of diseases, including cancer [8–10], cardiovascular diseases and metabolic disorders [11–15], and neurodegenerative diseases [16–18]. Also, due to notable features such as stability, high abundance in body fluids, and high cell- and tissue-specificity, circRNAs exhibit great potential to serve as biomarkers for diseases [19–22].

In the present review, we will briefly introduce the biology of circRNAs, including the biogenesis process, classification, and characteristics. Given that the biological function and mechanisms of gene regulation of circRNAs are not fully understood, we will summarize what has been widely acknowledged. In addition, since several features of circRNAs, including circular conformation, relatively low abundance, and overlap in sequence with other RNA counterparts, often hinder the investigation of circRNAs, we will then describe recent progress in computational strategies for identification and prediction of circRNAs. In contrast to benchmarking the strategies, we aim to give the readers a board introduction of circRNA biology and computational method, which will help them in designing their future studies and analyzing results. Readers interested in specific topics should refer to the reviews on circRNAs that have been summarized elsewhere [23–27].

## 2. Discovery of circRNAs

circRNAs were initially discovered via electron microscopy as a viroid in the mid-70s, because of the circular conformation [28, 29]. The biological analysis found that these circRNAs show several features, including (1) single-stranded, (2) high thermal stability, (3) self-complementarity in a rod-like structure, and (4) covalently closed as a loop [28]. Later, in the 90s, owing to advancement in computational biology and RNA sequencing, researchers finally determined the structure of the previously identified transcripts that show an inverted order of exons that is distinct from genomic DNA, which was mistakenly recognized as RNA splicing errors [30]. This study found that, although these transcripts are nonpolyadenylated and not as abundant as in a normal transcript, they are stable molecules and expressed in the cytoplasmic part of the cells [30].

The breakthrough in high-throughput sequencing (HTS) technology in the 21st century made it possible to deepen our understanding of circRNA sequences and functionality. In 2012, using deep RNA sequencing (RNA-seq) of normal and cancer stem cells from human samples, circRNAs were identified from a substantial fraction of spliced precursor message RNAs (pre-mRNAs) that showed a noncanonical order [1], suggesting a new feature of the gene expression program in human cells. Later, a close examination of circRNAs using Circle-Seq found that these molecules usually consist of up to five exons; however, each of them can be three times longer than the average expressed exon [31, 32]. A computational strategy was developed to specifically detect circRNAs, enabling identification of thousands of stable circRNAs [32]. As a proof-of-concept, using biochemical, functional, and computational analyses, this study showed that CDR1as, a known human circRNA, can bind miR-7 in neuronal tissues to function as a negative regulator [32].

Interestingly, treating RNAs with RNA exonuclease to deplete linear RNAs, researchers were able to perform bioinformatic analysis to identify complementary ALU repeats in introns; the results showed that circRNAs are abundant and stable RNA splicing products and are not randomly produced, suggesting that circRNAs are truly involved in gene expression regulation [31]. It is worth noting that all these discoveries would not have been made possible without the advancement of HTS technology.

## 3. Characterization of circRNAs

Thanks to the efforts from a number of research groups, to date, more than 20,000 different circRNAs have been identified, showing an unprecedented diversity of circRNAs among different species [33]. In addition, tissue and subcellular expression are also characterized. Surprisingly, in mammalians, most circRNAs are found in the brain, mainly in neuronal and synaptic functions [34, 35]. *In situ* sequencing was used to reveal the subcellular localization of circRNAs in the brain and found that as predicted, circRNA transcripts are enriched in the cytoplasm. However, nuclear localization was also found, though to a less extent [36]. Other studies also showed the role of circRNAs to regulate gene expression in the nucleus [4]. In other tissue types, such as the liver, heart, placenta, and blood, circRNAs are also found [36]. Another study not only investigated tissue-specific expression pattern but also explored the role of circRNAs in a development stage-specific manner and found that similar to adult human tissues, fetal tissues show an abundance of circRNAs [37].

Before we discuss the classifications of circRNAs, we will briefly introduce the noncoding RNA (ncRNA) family. As its name suggests, ncRNA is an RNA that is not translated into a protein. ncRNAs mainly consist of transfer RNA, ribosomal RNA (rRNA), and many other small RNAs such as long noncoding RNA (lncRNA: ≥200 nt), small noncoding RNA (sncRNA: 100-200 nt), miRNA (20-24 nt), and endogenous small interfering RNA (endo-siRNA). circRNAs have been categorized as ncRNAs; however, recent new studies challenged this view by demonstrating that circRNAs can code for proteins (Figure 1(d)) [38–40]. These studies showed that a group of circRNAs termed ribo-circRNAs, because they are associated with translating ribosomes, are bound by membrane-associated ribosomes, suggesting the existence of unexplored modes of regulation of genes and proteins [38, 39]. Another study showed that translation of circRNAs could be driven by m6A, the most abundant RNA modification [41]. Nevertheless, the characterizations of circRNAs have just started.

Stability is one of the distinct characteristics of circRNAs separating them from linear RNAs. In general, compared to linear RNAs, circRNAs are quite stable, because the lack of a poly(A) tail in circRNAs can protect them from exonuclease-mediated degradation [31]. This feature has been utilized to a recent engineering study to generate exogenous circRNAs, thus obtaining more potent and durable proteins in eukaryotic cells [42].

## 4. Biogenesis of circRNAs

**Figure 2:**
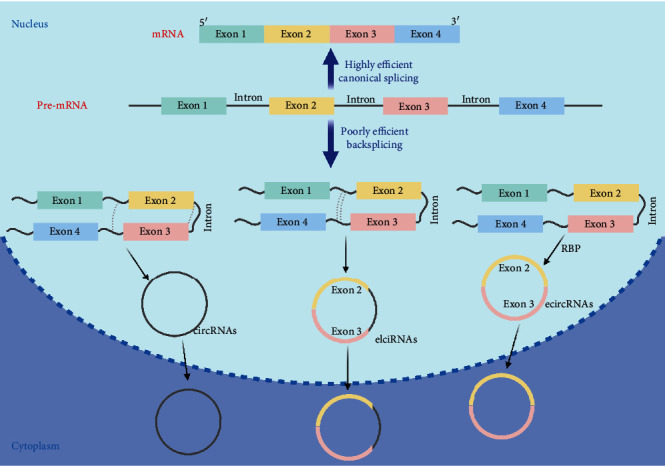
Biogenesis of circRNAs. Pre-mRNAs go through two splicing pathways to generate a linear RNA via highly efficient canonical splicing (top) or to produce circRNAs and an alternatively spliced linear RNA via poorly efficient backsplicing. As a result, different types of circRNAs can be produced (see discussion in text). Illustration was generated using BioRender.

Linear RNAs usually terminate with 5′ caps and 3′ tails and undergo canonical splicing; however, due to the closed loop structure, neither 5′-to-3′ polarity nor poly(A) tail can be found in circRNAs. Thus, circRNAs show stability over linear RNAs [31, 32]. Canonical splicing in pre-mRNAs is catalyzed by a spliceosome assembly, resulting in a linear RNA transcript with a 5′-to-3′ polarity. This splicing strategy is considered as highly efficient. Different from canonical splicing, circRNAs are generated via backsplicing, which, on the contrary, is considered as a noncanonical way (Figure 2). When the upstream 3′ splice acceptor site joins with a downstream 5′ splice donor site, the junction site is ligated by a 3′-5′ phosphodiester bond, resulting in covalently closed circRNAs. The sizes of mature circRNAs have a wide range from ~100 nt to 4 kb [43]. In human cells, the most common size is several hundred nucleotides spanning two or three exons [31, 44, 45]. Besides, long flanking introns comprising inverted repeat sequences have been proved to promote exon circularization [46, 47]. Unlike canonical splicing, backsplicing is usually considered as poorly efficient by approximately 1-3% of the former [48, 49].

## 5. Categories of circRNAs

The RNA research community has annotated four different types of alternative splicing, including (1) intron retention, (2) exon skipping, (3) alternative 5′ splicing, and (4) alternative 3′ splicing [50], suggesting the complexity of the biogenesis of circRNAs. Based on these four types of alternative splicing, circRNAs can be categorized into four types: intron-derived circRNAs, exon-derived circRNAs (ecircRNAs), intergenic circRNAs, and exon-intron circRNAs (elciRNAs) [51]. Among these types, ecircRNAs are predominantly generated from backspliced exons as the largest type of circRNAs, accounting for the majority of the circRNAs that have been discovered.

## 6. Major Biological Function and Disease Relevance

In contrast to mRNAs and miRNAs, the biological functions of circRNAs are largely unclear. However, in the last decades, a number of seminar investigations have been conducted to demonstrate a wide variety of roles that circRNAs might play. Here, we briefly summarize some critical functions that circRNAs are implied to play.

CircRNAs can act as miRNA sponges, that is to say, by its name, circRNAs are reservoirs of miRNAs (Figure 1(a)). It is well known that miRNAs belong to a family of ncRNAs that regulate gene expression in a wide range of biological processes. The current view of circRNAs as a miRNA or protein sponge is that circRNAs regulate miRNA activity, thus modulating the expression of miRNA target genes [52]. As illustrated in Figures 1(b) and 1(c), in healthy and tumor tissues, specific circRNAs harbor miRNAs that target different types of genes such as tumor-suppressor genes or oncogenes, thus exhibiting various biological effects. Owing to the importance of miRNAs that bind to circRNA sponges, miRNA-based computational pipelines have been established to predict circRNA targets. We will revisit this topic in a later section of this review. In addition to regulating miRNA, circRNAs also serve as the sponge of RNA-binding proteins (RBPs) to regulate intracellular transport (Figure 1(a)), thereby modulating gene expression of relevant RBPs of interest [53]. Readers with interests in this topic could find more details in several recent reviews [54–56]. As shown in Figure 1(a), circRNAs, such as ciRS-7, also bind to Argonaute (AGO) proteins in a miR-7-dependent manner [57], which could regulate mRNA transcription and translation.

A number of circRNAs have been identified as miRNA sponges. A prominent example is ciRS-7, which serves as a miR-7 sponge [32, 57]. ciRS-7 is highly expressed in the cytoplasm and has more than 70 miR-7 target sites [57]. It has been reported that ciRS-7 functions as both tumor-suppressor and oncogenic sponges, serving as a promising biomarker for various cancers such as colorectal cancer [58], hepatocellular carcinoma [59], esophageal squamous cell carcinoma [60, 61], cervical cancer [62], and pancreatic cancer [63]. Interestingly, some studies also show that ciRS-7 promotes *β*-amyloid precursor protein (APP) and *β*-site APP-cleaving enzyme (BACE1) degradation [16]; thus, it might also play a role in Alzheimer's disease.

**Figure 3:**
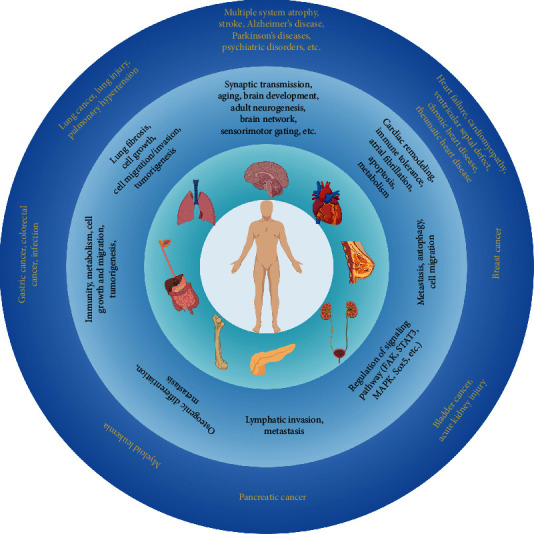
circRNAs and human diseases. circRNAs are abundantly expressed in various tissues and are implicated in a number of human diseases, including cancer and brain disorders. Illustration was generated using BioRender.

CircRNAs have been implicated in several diseases such as cancer, cardiovascular diseases, and neurodegenerative diseases. First, circRNAs are abundantly and dynamically expressed in the brain [35] and have been shown to participate in a variety of brain-related processes such as synaptic transmission [64], aging [65–67], sensorimotor gating [64], cell-type-specific interaction and brain network [68, 69], development and adult neurogenesis [70–73]. Therefore, many investigators have demonstrated that circRNAs play important roles in the pathogenesis of a number of brain-related disorders such as multiple system atrophy [74], transient focal ischemia and stroke [75–82], neuropsychiatric disorders [64, 68], Alzheimer's disease [83, 84], Parkinson's disease [85], brain bacterial infection [86], brain tumors [87], and metabolic diseases [88]. Second, circRNAs are also highly expressed in the heart [89] and are reported to play a role in cardiac remodeling [90], stress response [91], endothelial-to-mesenchymal transition [92], metabolism [93, 94], immune tolerance [95], atrial fibrillation [96], and apoptosis [97]. Thus, these circRNAs are suggested to participate in heart diseases such as heart failure [91, 97, 98], ventricular septal defect [99], chronic heart disease [92, 100–102], alcoholic cardiomyopathy [93], and rheumatic heart disease [96]. In the lung, circRNAs have been found to be important in lung fibrosis [103], cell growth [104], cell migration and invasion [105–108], cancer tumorigenesis [109], and so on; thus, these circRNAs play a role in various lung-related diseases such as lung cancer [105, 107, 110–112], lung injury [113], and pulmonary hypertension [114]. The role of circRNAs in various human diseases are annotated in Figure 3.

## 7. Bioinformatic Analysis of circRNAs

Given the importance of circRNAs in gene expression regulation, a growing interest emerges in identifying novel circRNAs and understanding their biological functions. Therefore, genome-wide identification and prediction of circRNAs are crucial for the study of circRNA biological functions [115, 116].

Effective investigation of circRNAs highlights a particular need of HST technology. In the past years, the high-throughput microarray was a dominating means to study the junction sequences of circRNAs [117, 118]. By designing probes to target specific circular junction sites, a circRNA microarray allows accurate and reliable detection of individual circRNAs. Following a detailed annotation of potential miRNA target sites, a circRNA microarray helps to reveal their potential roles as a miRNA sponge. The isolated RNA samples go through a pretreatment process, in which RNase R is used to remove linear RNAs and improve the purity of circRNAs. However, the limited number of known circRNAs during annotation and the use of a junction sequence to identify circRNAs bring limitations to the application of a microarray. Therefore, in recent years, high-throughput RNA-seq technology has become the dominant approach to identify circRNAs. As a result, a number of computational pipelines for circRNA identification have been developed to identify circRNAs from massive RNA-seq databases.

**Figure 4:**
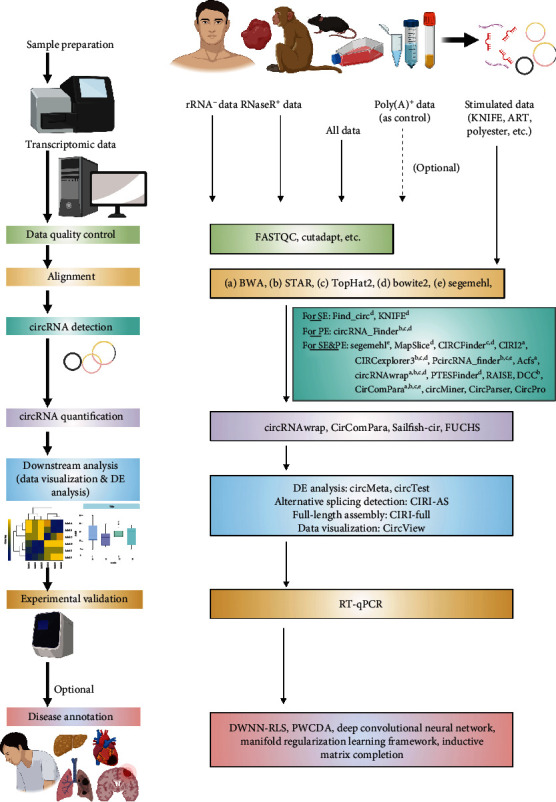
Key steps in studying circRNAs using publicly available pipelines. For read data, the library preparation is similar to traditional mRNA extraction. For stimulated data, several tools such as KNIFE and CIRI-simulator can be used. Alignment methods for linear RNAs, such as STAR and TopHat, are also commonly used for circRNAs, Therefore, a number of professional pipelines shown in Table 1 can be applied for circRNA detection, such as DCC and CIRI. For downstream analysis, other optional pipelines can be employed for different purposes. Finally, several pipelines can be used to check the association of circRNAs and diseases. The authors apologize for omitting any key pipelines or key steps. Illustration was generated using BioRender.

In this section, we introduce several commonly used computational pipelines for the identification of circRNAs.  Figure 4 outlines several key steps in studying circRNAs using publicly available pipelines; thus, readers could have a brief idea of where to choose individual pipelines. We apologize for omitting any key pipelines or key steps. Thus, we highly recommend readers to refer to other reviews specifically on this topic [119–122].  Table 1 provides a comprehensive summary of online tools for the study of circRNAs, while Table 2 is a list of computational pipelines for optional analysis of circRNAs. To our knowledge, this is the most comprehensive and updated summary of circRNAs tools. In addition, a video-based introduction to the identification of circRNAs from RNA-seq is also available from JOVE [123].

To effectively identify circRNAs, no matter which computational pipeline is used, one needs to discriminate circRNAs from linear RNAs. Several biochemical assays have been developed to distinguish circRNAs from other backsplicing products, including (1) divergent primer PCR, (2) relative migration of circRNAs from a canonical linear RNA in an agarose gel, (3) 2D gel electrophoresis, (4) gel trapping, and (5) exonuclease enrichment [119]. Other than biochemical enrichment strategies, deep sequencing with novel bioinformatics analysis has been developed to perform a comprehensive characterization of circRNAs. To date, candidate- or pseudo-reference-based strategies have been designed in computational pipelines [119, 124]. The candidate-based strategy uses a list of candidate junctions that were generated from previous models [1, 119]. Thus, this approach is able to analyze rRNA-depleted libraries in a fast manner; however, it has an obvious limitation in unannotated transcripts. Constructing putative circRNA sequences with gene annotation, a pseudo-reference-based approach, such as KNIFE [45], NCLScan [125], and PTESFinder [126], has become widely used. These approaches use several systematic filtering steps to remove false positive [120, 124]. For example, by using PTESFinder to analyze previously mined RNA-seq reads, significantly more distinct structures were found than previously reported (between 13% and 42%), whereas a significant number of reads were excluded by PTESFinder due to low map quality or multiple map locations [126]. Thus, owing to these novel pipelines, the highest specificity and sensitivity could be achieved. In addition to these strategies, a fragmented-based strategy is also frequently used, in which a backsplicing junction is aligned to the genome [120].

Although these detection pipelines could significantly accelerate the identification of novel circRNAs, inconsistency in results might occur when switching from one pipeline to another. Thus, evaluations for different circRNA pipelines had been performed. A recent comparison study has provided a comprehensive and unbiased comparison among several circRNA detection pipelines [120]. This study used a number of measurements to evaluate their performance, including precision, sensitivity, F1 score and area under curve, random access memory consumption, running time, and physical disk space utilization, and concluded that CIRI, CIRCexplorer, and KNIFE have better performance [120]. An earlier review had summarized the criteria in different pipelines or algorithms to perform filtering and accuracy evaluation; thus, we highly recommend readers to refer to this review [127]. In addition, it is worth noting that, as many studies have already pointed out, this study also suggested that no individual pipeline could achieve the best performance among all the metrics used, indicating an urgent need to refine and integrate all these available methods for circRNA detection [128, 129]. For the time being, pairing different pipelines possibly produces a much more reliable output, for example, circRNA and find_circ.

To increase the accuracy in circRNA identification, two concerns should be kept in mind when designing experiments: (1) At the experimental stage, many variations can affect circRNA abundances, such as RNA purification, size selection, and RNA fragmentation followed by adaptor ligation. RNase R is commonly used to digest linear RNAs to enrich circRNAs for sequencing, but not all circRNAs are resistant to RNase R; conversely, a few linear RNAs can avoid RNase R digestion. (2) A small fraction of circRNAs inherently exits in common cell lines, which account for approximately 2-4% of the total mRNAs. This level is higher in platelets. Therefore, significant biases will arise when bioinformatic analysis relies on junctional reads. As a result, a high rate of false positives occurs. To this end, most pipelines apply multiple high thresholds on absolute read counts. Other pipelines employ statistical approaches to reduce the reliance on the thresholds.

The current limitations of circRNA research include limited methods available to detect and quantify circRNAs. Although RT-qPCR-based methods are low cost and highly sensitive methods that can be easily applied in many laboratories, they are not high-throughput methods that can detect and quantify circRNAs. While RNA-seq has served as the main method that has high sensitivity and high throughput, the cost can be high, and it usually requires sufficient computational power. A detailed comparison of different methods for circRNA detection and quantification can be found elsewhere [7]. The genome-wide prediction tools, as discussed here, can largely assist in the identification and characterization of circRNAs; however, it is still challenging to assess the circRNA-miRNA and circRNA-protein interactions. In most cases, the sequences of the circRNAs are not clear, which might be problematic for downstream analysis such as miRNA target prediction. In addition, bioinformatic analysis relying on reads spanning the backsplice junction could be problematic because of the biases in read density [127, 130]. We envision that future studies could help solve these critical issues.

In sum, although a number of pipelines are available for circRNA research, how to obtain genome-wide detection of circRNAs with high sensitivity and specificity remains a challenge. It is foreseeable that in the future, a comprehensive comparison of these pipelines, as well as a comparison in computational power using publicly available datasets, will become available.

## 8. Comprehensive Databases of circRNAs

Other than the computational tools that are used for the detection and identification of circRNAs, it is undoubtedly important that a comprehensive understanding of the association of these identified circRNAs and human diseases is eagerly expected. Therefore, several circRNA databases have been established containing thousands of mammalian circRNAs carefully selected from various sources. Thus, detailed information, such as genome sequence, subcellular location, and disease annotation, are all provided to researchers working on circRNAs.

Table 3 summarizes the most updated circRNA databases that are publicly available. Among these databases, several of them are widely used, such as Circ2Traits [131], circBase [132], and circFunBase; they are among the earliest circRNA databases that are commonly used. Here, we briefly discuss how to make full use of CircBase. We suggest that readers find more useful information from other papers [120, 132, 133].

**Figure 5:**
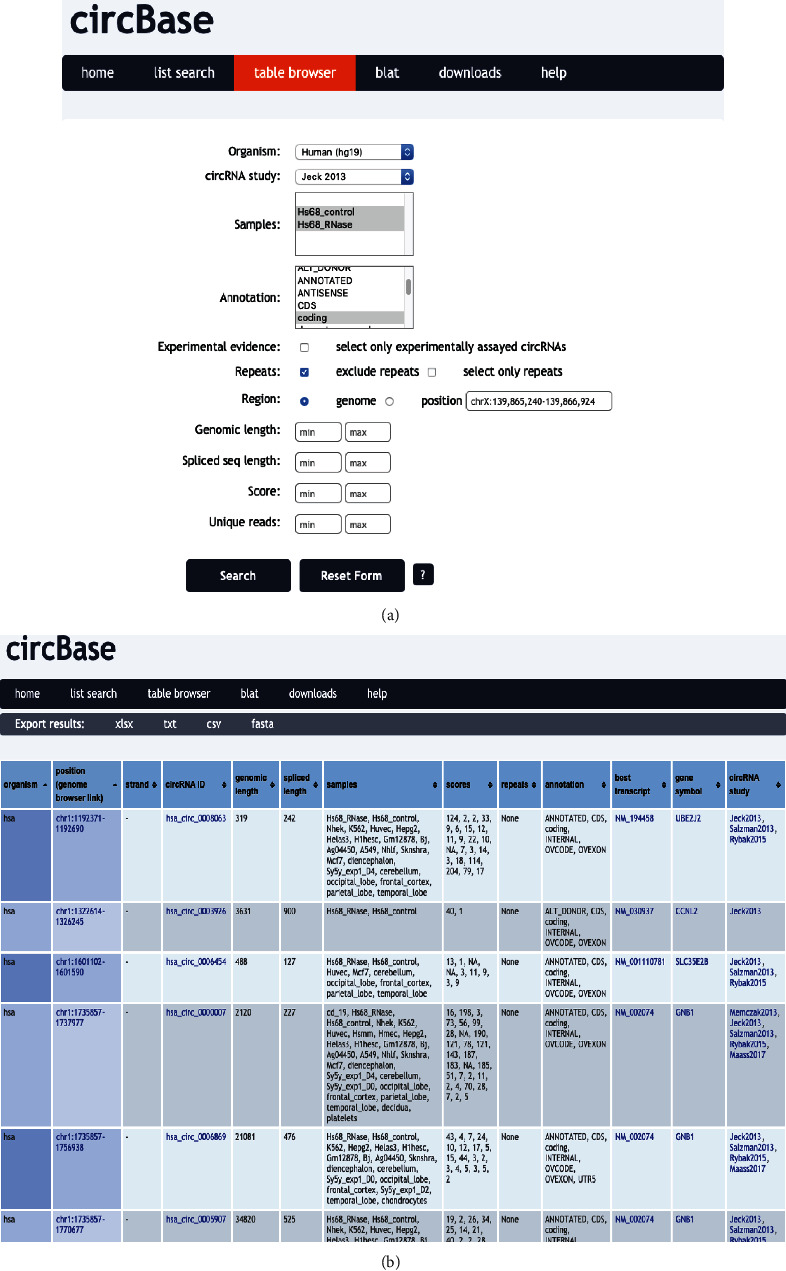
Searching circRNAs with circBase table browser. This illustration gives a brief introduction on how to search circBase using the table browser option. (a) circBase table browser interface. (b) An output from the result page after submitting queries.

circBase, as one of the earliest developed databases for circRNAs, was brought in 2014 and has been widely used in the circRNA community [132]. As of today, the original report of circBase has been cited for nearly 600 times, indicating that it has been regarded as a powerful tool for the community [132]. The main aim of developing circBase was to provide summary information of individual circRNAs that have been identified, together with their genomic context. Three ways of searching circBase were provided, including simple search, list search, and table browser search. These searching methods can be easily found on the main page of the website (http://www.circbase.org/). Simple search, with identifiers, genomic coordinates, sequences, gene ontology identifiers, transcript ID, and gene symbols, is the easiest way of searching the database. List search gives users an option to paste or upload a list of several circRNAs or refseq identifiers, as well as gene symbols. Organism is required to be selected. Table browser search is a quick search option based on the browser interface. Note that organism and dataset information is required to be selected. As illustrated in Figure 5(a) as an example, in the circBase table browser page, users could select human as Organism and use a dataset from a previous study [31]. Both sample conditions and annotation allow for multiple selection. After submitting using the search button, a detailed result page will be returned, with basic information on individual circRNAs that matches the query (Figure 5(b)). The listed information includes organism data source; genomic position information which directs to a link from the UCSC genome browser, with full information on strand; circRNA ID; genomic length; spliced length; list of samples that contain the circRNAs; and number of reads. By clicking a single circRNA, the link will direct the users to a single record page, which contains detailed information on a particular circRNA. Detailed information on how to use circBase can be found on the documentation page (http://www.circbase.org/doc/help_mod.html). In addition, in circBase, data can be exported in standardized formats such as xlsx, txt, csv, or fasta, providing users a variety options to integrate with other analysis tools. In general, circBase is an excellent database that focuses on elementary information of backsplicing junction coordinates.

CircBase has been used in several key studies to identify targeted circRNAs, and this identification can be validated by quantitative real-time PCR or downstream analysis. Thus, circBase plays an important role in the identification of potential biomarkers for various cancers [134–143].

Here, we are giving the readers another example, circad (circRNAs associated with diseases), a database mainly for disease-associated circRNAs [144]. After submitting a circRNA's name in the browser (http://clingen.igib.res.in/circad/), the database returns with a selection of different organisms. Selecting one organism will bring users to the next page, which has information including genome locus, gene name, disease association, fold change, and a publication's PubMed ID (PMID). It is worth noting that as an exception to many databases, circad includes detailed information of the primers used in that publication. A detailed documentation on how to use circad can be found (http://clingen.igib.res.in/circad/img/circad.pdf).

In addition, several other databases have also been developed. Here, we provide a brief introduction to each of them (for the web links and last updated data, as well as references for individual databases, please refer to Table 3):

1. *circ2Traits* is the first comprehensive database of potential disease association of circRNAs in humans [131]; in this database, users can find SNPs associated with diseases and AGO interaction sites
2. *SomaniR* is a database mainly for cancer somatic mutation in miRNAs and their target sites that might potentially interact with circRNAs [145]
3. *CircNet* is a database with resources of novel circRNAs, integrated miRNA-target network, expression, annotations, and sequences of circRNA isoforms [146]
4. *circRNADb* is a human circRNA database that contains more than 32k annotated exonic circRNAs [147]
5. *TSCD* is an integrated tissue-specific circRNA database, which deposits features of tissue-specific circRNAs [148], and users could find tissue-specific expression in both mouse and human adult and fetus
6. *CIRCpedia*, a 2nd version of this database, is based on CIRCpedia, for comprehensive circRNA annotation from >180 RNA-seq datasets [149]; this database contains circRNA annotations across 6 species, including human, mouse, rat, zebrafish, fly, and worm
7. *CircR2Disease* is a manually curated database that gives users a comprehensive resource for circRNA deregulation in diseases [150]; it contains >700 associations between 661 circRNAs and 100 diseases so that users can study the mechanism of disease-related circRNAs
8. *exoRBase* is a database that has >58k circRNAs in human blood exosomes, which helps users to identify exosomal biomarkers [151]
9. *TRCirc* can be used to study transcriptional regulation of circRNAs based on ChIP-seq and RNA-seq results [152]; it also enables analysis of methylation level
10. *CircRNAdisease* is another newly developed database to understand circRNA and disease associations [153]; it contains 354 associations between 330 circRNAs and 48 diseases
11. *CircBank* is a comprehensive database for human circRNAs, and it contains 5 features such as a miRNA binding site, conservation of circRNAs, m6A modification, mutation, and protein-coding potential of circRNAs [154]; note that this database has a novel naming system for circRNAs
12. *circFunbase* is a database featured by a high-quality functional circRNA resource [155]; most of the resource has been validated by experiments; it contains circRNAs from a wide variety of species, such as plants and animals (human, monkey, rat, mouse, etc.)
13. *LncACTdb* is a database mainly for endogenous RNAs such as circRNAs in different species and diseases [156]; it contains about 60 experimentally supported circRNA interactions
14. *CropCircDB* is a database specifically for crop circRNAs such as maize and rice [157]; it also has validated crop circRNAs in response to abiotic stress
15. *AtCircDB* is another plant-specific database mainly for *Arabidopsis* circRNAs [158]
16. *MiOncoCirc* is a database that contains circRNAs from cancer cell lines and tumor samples [23]
17. *Circad* is another disease-associated database for circRNAs [144]; it has >1300 circRNAs implicated with 150 diseases; besides, it has circRNAs from 5 species, including human, rat, and mouse
18. *ncrpheno* is a database mainly for ncRNAs; however, it contains 848 circRNAs as well as circRNA-related diseases [159]
19. *NPInter* (v4) is the 4th version of the NPInter database that integrates 6M newly identified ncRNA interactions including circRNA interactions [160]; it also contains circRNAs from dozens of species, including human, mouse and rat
20. *CircAtlas* (v2) is a database that integrated 1M circRNAs across 6 species, including human, macaca, mouse, rat, pit, and chicken as well as 19 normal different tissues [161]; it also describes a conservation score, coexpression, and regulatory networks
21. VirusCircBase is a comprehensive database of viral circRNAs [162]; it contains 12K circRNAs, most in viruses and infectious diseases

To our knowledge, this summary list is the most updated summary of circRNA databases. Here, we recommend the following principles for readers to choose each database based on the purposes of their experiment and analysis:

1. *Disease association*: for projects that are aimed at comparing several disease conditions, these databases could be chosen—circ2Traits, circR2Disease, circRNAdisease, Circad, ncrpheno, and CircAtlas
2. *Cross-species comparison*: for projects involving a cross-species comparison, these databases contain circRNA information on several different species, including CIRCpedia, circFunbase, Circad, NPInter (v4), and CircAtlas (v2)
3. *Transcriptomic regulation*: for projects that are aimed at studying epigenetic regulation of gene expression, these databases could be chosen—TRCirc, CircBank, LncACTdb, and NPInter (v4)
4. *Tissue-specific purpose*: for projects that are aimed at comparing circRNAs in a wide range of normal tissues. The databases that could fulfill this purpose are TSCD, NPInter (v4), and CircAtlas (v2). Nevertheless, it is advised to perform an initial search via exoRBase for a blood-related project, whereas VirusCircBase should be the first choice for a virus-related project. However, it is always preferable to go through each relevant database if necessary.

## 9. Concluding Remarks

In the past few years, growing evidence has been seen in circRNAs as potential diagnostic and prognostic biomarkers for human diseases. Because most circRNAs are abundantly expressed in a wide variety of tissue types and cell types, and that circRNAs show great stability and a robust regulation role in gene expression, circRNAs will become favorable biomarker candidates that are worthy of investigation in both basic and clinical medical sciences.

One of the bottlenecks in studying circRNAs is detection and identification from genome-wide datasets. The emerging field of big data enables us an unprecedented opportunity to store, manage, process, and analyze biological data that contains information with tremendous complexity. Therefore, a computational strategy that mainly uses publicly available pipelines and databases developed and shared by circRNA communities could enormously reduce the challenges and increase the efficiency of applying bioinformatics knowledge to identify key circRNAs that could bring diagnostic and prognostic values.

In this review, we have briefly introduced the biology of circRNAs, including characteristics, biogenesis, biological functions, and disease relevance, as well as several computational approaches that enable researchers to detect and identify potential novel circRNAs. Finally, we have highlighted several publicly available computational resources for the analysis of circRNAs that, to our knowledge, are the most completed and updated. Thus, we hope this review will help researchers at various levels in their current and future studies on circRNAs.

The study of circRNAs has just begun, and the field is relatively young. A number of outstanding questions are still waiting to be addressed, such as the association of circRNAs in disease progression and development, the value of circulating circRNAs to predict their abundance and relevance in deep tissues, novel functions of circRNAs beyond sponges for small molecules, and the efficiency of combining single-molecule HTS technology with circRNAs [34, 51, 119, 163]. Nevertheless, the continuous efforts in detection, identification, and characterization of circRNAs will lead to our understanding of circRNAs' function and clinical value into a completely new lever.

## Figures and Tables

**Table 1:** A comprehensive list of available pipelines and databases for detection, identification, and evaluation of circRNAs.

Name	Year	Web functions	Web links	Last updated	Refs.
MapSlice 2	2010	A highly accurate algorithm for alignment of RNA-seq reads to splice junction	http://www.netlab.uky.edu/p/bioinfo/MapSplice2	06-13-2016	[165]
Find_circ	2013	To detect backspliced sequencing reads, which is an indication of circRNAs in RNA-seq data	https://github.com/marvin-jens/find_circ	01-06-2017	[32]
CIRCfinder	2013	A pipeline to map junction reads for intronic circRNAs	https://github.com/YangLab/CIRCfinder	03-25-2016	[166]
CircRNA_finder	2014	A pipeline to search for circRNAs from RNA-seq data	https://github.com/orzechoj/circRNA_finder	10-14-2019	[167]
CIRI	2015	A *de novo* circRNA identification tool to detect circRNAs from transcriptome data	https://sourceforge.net/projects/ciri/ https://omictools.com/ciri-tool https://sourceforge.net/projects/ciri/	04-16-2020	[168, 169]
KINFE	2015	Known and Novel IsoForm Explorer is a statistically based splicing detection tool for circRNAs and linear isoforms from RNA-seq data	https://github.com/lindaszabo/KNIFE	07-14-2017	[45]
miARma-seq	2016	A comprehensive tool for the identification of miRNAs, mRNAs, and circRNAs	http://miarmaseq.cbbio.es/ (not accessible) https://sourceforge.net/projects/miarma/	06-15-2018	[170]
NCLscan	2016	A stepwise alignment strategy to eliminate false calls; to identify both intragenic and intergenic NCL transcripts from paired-end RNA-seq data	https://github.com/TreesLab/NCLscan ftp://treeslab1.genomics.sinica.edu.tw/NCLscan	05-21-2020	[125]
UROBORUS	2016	A computational pipeline to detect circRNAs in total RNA-seq data	http://uroborus.openbioinformatics.org/ (NA)	NA	[171]
deepBase v2.0	2016	To decode evolution, expression patterns, and functions of diverse ncRNAs (incl. circRNAs) across 19 species	http://biocenter.sysu.edu.cn/deepBase/ (NA) https://omictools.com/deepbase-tool	NA	[172]
Acfs	2016	A pipeline for de novo circRNA identification, allowing the discovery of circRNAs from RNA-seq data	https://github.com/arthuryxt/acfs	02-16-2017	[173]
pcircRNA_finder	2016	A pipeline for the prediction of plant circRNAs	http://ibi.zju.edu.cn/bioinplant/tools/manual.htm	NA	[174]
PTESFinder	2016	A computational pipeline for identifying posttranscriptional exon shuffling events from HTS data	https://sourceforge.net/projects/ptesfinder-v1/	09-04-2017	[126]
CirComPara	2017	A multimethod comparative bioinformatics pipeline to detect and study circRNAs from RNA-seq data	https://github.com/egaffo/CirComPara	12-21-2016	[172]
RAISE	2017	A pipeline for circRNA backsplice sites and used for identification, quantification of abundance, and prediction of their internal structure using RNA-seq data	https://github.com/liaoscience/RAISE	03-13-2017	[175]
CircPro	2017	An integrated tool for identification of circRNAs with protein-coding potential	http://bis.zju.edu.cn/CircPro/	NA	[176]
AutoCirc	2017	A pipeline that allows definition of m6A circRNAs with cell-type-specific expression	https://github.com/chanzhou/AutoCirc	04-08-2020	[177]
PRAPI	2018	A pipeline to analyze differential expression of circRNAs	http://forestry.fafu.edu.cn/tool/PRAPI/	12-05-2019	[178]
hppRNA	2018	A snakemake-based RNA-seq pipeline to analyze circRNAs	https://sourceforge.net/projects/hpprna/	12/18-2018	[179]
NetMiner	2018	A pipeline to construct a high-quality RNA-seq-based gene coexpression network and to predict biological functions of novel circRNAs	https://github.com/czllab/NetMiner	11-06-2017	[180]
Docker4Circ	2019	A comprehensive analysis of circRNAs in human and model organisms, including circRNA prediction, classification, and annotation	https://github.com/kendomaniac/docker4seq https://github.com/mbeccuti/4SeqGUI	02-20-2019 07-21-2017	[181]
circtools	2019	A Python-based framework for circRNA-related tools that use a single command to unify different functionalities	https://github.com/dieterich-lab/circtools	02-27-2019	[182]
CircRNAFisher	2019	For de novo genome-wide circRNA identification and annotation	https://github.com/duolinwang/CircRNAFisher. Note: bowtie2 is required. http://bowtie-bio.sourceforge.net/bowtie2	07-06-2019	[183]
CIRCexplorer3	2019	Analysis of circRNAs and linear RNAs from rRNA-depleted RNA-seq data	https://github.com/YangLab/CLEAR https://github.com/YangLab/CIRCexplorer2	01-28-2020	[121, 184]
circCode	2019	A Python-based pipeline to identify the coding ability of circRNAs	https://github.com/PSSUN/CircCode	05-19-2020	[185]
circMiner	2020	A fast and sensitive tool to detect circRNAs from RNA-seq data	https://github.com/vpc-ccg/circminer	05-01-2020	[186]
circDeep	2020	A deep learning approach for circRNA classification from long noncoding RNAs	https://github.com/UofLBioinformatics/circDeep	01-28-2018	[187]
circDBG	2020	circRNA detection from high-throughput sequence data with de Bruijn's graph	https://github.com/lxwgcool/CircDBG	03-06-2020	[188]
CircParser	2020	A Unix/Linux-based pipeline to predict host gene circRNAs	https://github.com/SharkoTools/CircParser	03-27-2020	[189]

NA: not applicable. Last access date: 05-25-2020.

**Table 2:** Summary of computational pipelines for optional analysis of circRNAs.

Name	Year	Description	Web links	Last updated	Refs.
CircTest	2016	Test the variation of circRNAs in respect to host genes	https://github.com/dieterich-lab/CircTest	01-04-2016	[190]
CIRI-AS	2016	Alternative splicing detection	https://sourceforge.net/projects/ciri/files/CIRI-AS	07-04-2016	[191]
sailfish-cir	2017	Quantification using model-based framework	https://github.com/zerodel/Sailfish-cir	05-04-2017	[192]
FUCHS	2017	Towards full circRNA characterization	https://github.com/dieterich-lab/FUCHS	09-28-2017	[193]
CircRNAwrap	2019	Transcript prediction and abundance estimation	https://github.com/liaoscience/circRNAwrap	04-19-2019	[194]
CIRI-full	2019	Full-length assembly	https://sourceforge.net/projects/ciri/	04-16-2020	[195]
circMeta	2020	Genomic feature annotation, differential expression analysis for circRNAs	https://github.com/lichen-lab/circMeta	10-01-2019	[196]
CIRIquant	2020	Quantification and differential expression analysis	https://sourceforge.net/projects/ciri/files/CIRIquant/	04-16-2020	[197]

NA: not applicable. Last access date: 05-25-2020.

**Table 3:** A list of web databases for circRNA studies.

Name	Year	Functions	Database links	Last updated	Refs.
Circ2Traits	2013	A database for circRNAs with potential disease association, observed from GWAS-associated SNPs and potential interactions with miRNAs	http://gyanxet-beta.com/circdb/ https://github.com/shaoli86/circ2Traits	11-18-2019	[131]
circBase	2014	A web server-based database for circRNAs	http://circbase.org/	12-15-2015	[132]
SomaniR v2.0	2016	A database of cancer somatic mutation in miRNA and target sites to interact with miRNAs or circRNAs and mRNAs	http://compbio.uthsc.edu/SomamiR/	NA	[198]
CircNet	2016	A database providing resources of novel circRNAs, integrated miRNA-target network, expression, annotations, and sequences of circRNA isoforms	http://circnet.mbc.nctu.edu.tw/ (NA) https://omictools.com/circnet-tool	NA	[146]
circRNADb	2016	A circRNA database containing >32k human exonic circRNAs	http://reprod.njmu.edu.cn/cgi-bin/circrnadb/circRNADb.php	03-03-2016	[147]
TSCD	2017	An integrated database (tissue-specific circRNA database) to deposit features of tissue-specific circRNAs	http://gb.whu.edu.cn/TSCD/	NA	[148]
CIRCpedia v2	2018	An updated database for comprehensive circRNA annotation from >180 RNA-seq datasets	https://www.picb.ac.cn/rnomics/circpedia/ https://www.picb.ac.cn/rnomics/circpedia_v1/	07-07-2018	[149]
CircR2Disease	2018	A curated database for circRNAs which is experimentally supported	http://bioinfo.snnu.edu.cn/CircR2Disease/ (NA)	03-31-2018	[150]
exoRBase	2018	A database of circRNAs, lncRNAs, and mRNAs from RNA-seq data of human blood exosomes	http://www.exorbase.org/	07-2017	[151]
TRCirc	2018	A database for providing information on transcriptional regulation of circRNAs	http://www.licpathway.net/TRCirc/view/index	NA	[152]
CircRNAdisease	2018	A curated database of circRNAs and disease association	http://cgga.org.cn:9091/circRNADisease/	04-27-2018	[153]
Circbank	2019	A database of human circRNAs from different sources	http://www.circbank.cn/	NA	[154]
CircFunBase	2019	A database with functional circRNA resources including predicted functions that have been experimentally validated	http://bis.zju.edu.cn/CircFunBase/	10-08-2019	[155]
LncACTdb 2.0	2019	A database of endogenous RNAs including circRNAs	http://www.bio-bigdata.net/LncACTdb/	NA	[156]
CropCircDB	2019	A circRNA database for crops in response to abiotic stress	http://genome.sdau.edu.cn/crop/ http://deepbiology.cn/crop/	05-10-2018	[157]
AtCircDB	2019	A tissue-specific database for *Arabidopsis* circRNAs	http://genome.sdau.edu.cn/circRNA (NA)	NA	[158]
MiOncoCirc	2019	A most comprehensive database of cancer-based circRNAs, providing a reference of circRNAs from cancer cell lines and tumor tissues	https://mioncocirc.github.io/	NA	[23, 199]
Circad	2020	A curated database of circRNAs associated with diseases	http://clingen.igib.res.in/circad/	NA	[144]
ncrpheno	2020	A database that integrates and annotates ncRNA-disease association data	http://lilab2.sysu.edu.cn/ncrpheno (NA)	NA	[159]
NPInter v4	2020	An integrated database of nvRNA interaction, including circRNA interaction	http://bigdata.ibp.ac.cn/npinter4	09-2019	[160]
CircAtlas 2.0	2020	An integrated database that contains >1 million highly reliable circRNAs in vertebrates	http://circatlas.biols.ac.cn/	03-30-2020	[161]
VirusCircBase	2020	A database of virus circRNAs, providing fundamental atlas for further study	http://www.computationalbiology.cn/ViruscircBase/home.html	01-30-2020	[162]

NA: not applicable. Last access date: 05-25-2020.

## Abbreviations

AGO: Argonaute protein
APP: *β*-Amyloid precursor protein
BACE: *β*-Site APP-cleaving enzyme
circRNAs: Circular RNAs
ecircRNAs: Exon-derived circRNAs
elciRNAs: Exon-intron circRNAs
endo-siRNA: Endogenous small interfering RNA
HTS: High-throughput sequencing
lncRNA: Long noncoding RNA
lncRNA: Long noncoding RNA
ncRNA: Noncoding RNA
pre-mRNA: Precursor message RNAs
RBP: RNA-binding protein
RNA-seq: RNA sequencing
rRNA: Ribosomal RNA
sncRNA: Small noncoding RNA
tRNA: Transfer RNA.
